# Homologs of Human Dengue-Resistance Genes, *FKBP1B* and *ATCAY*, Confer Antiviral Resistance in *Aedes aegypti* Mosquitoes

**DOI:** 10.3390/insects10020046

**Published:** 2019-02-02

**Authors:** Seokyoung Kang, Dongyoung Shin, Derrick K. Mathias, Berlin Londono-Renteria, Mi Young Noh, Tonya M. Colpitts, Rhoel R. Dinglasan, Yeon Soo Han, Young S. Hong

**Affiliations:** 1 Emerging Pathogens Institute and the Department of Infectious Diseases and Immunology, College of Veterinary Medicine, University of Florida, Gainesville, FL 32611, USA; rdinglasan@epi.ufl.edu; 2 Florida Medical Entomology Laboratory, Department of Nematology and Entomology, Institute of Food and Agricultural Sciences, University of Florida, Vero Beach, FL 32962, USA; dshin@ufl.edu (D.S.); d.mathias@ufl.edu (D.K.M.); 3 Department of Entomology, College of Agriculture, Kansas State University, Manhattan, KS 66506, USA; blondono@ksu.edu; 4 Department of Agricultural Biology, College of Agriculture and Life Sciences, Chonnam National University, Gwang-ju 500-757, Korea; annemi@hanmail.net (M.Y.N.); hanys@chonnam.ac.kr (Y.S.H.); 5 Department of Microbiology, Boston University School of Medicine, National Emerging Infectious Diseases Laboratories, Boston, MA 02118, USA; tmcol@bu.edu; 6 Access Bio, Inc., Somerset, NJ 08873, USA

**Keywords:** *Aedes aegypti*, dengue virus type 2, anti-dengue viral gene, *AeFKBP1*, *AeATCAY*, RNA interference

## Abstract

Dengue virus (DENV) is transmitted by mosquitoes and is a major public health concern. The study of innate mosquito defense mechanisms against DENV have revealed crucial roles for the Toll, Imd, JAK-STAT, and RNAi pathways in mediating DENV in the mosquito. Often overlooked in such studies is the role of intrinsic cellular defense mechanisms that we hypothesize to work in concert with the classical immune pathways to affect organismal defense. Our understanding of the molecular interaction of DENV with mosquito host cells is limited, and we propose to expand upon the recent results from a genome-scale, small interfering RNA (siRNA)-based study that identified mammalian host proteins associated with resistance to dengue/West Nile virus (DENV/WNV) infection. The study identified 22 human DENV/WNV resistance genes (DVR), and we hypothesized that a subset would be functionally conserved in *Aedes aegypti* mosquitoes, imparting cellular defense against flaviviruses in this species. We identified 12 homologs of 22 human DVR genes in the *Ae. aegypti* genome. To evaluate their possible role in cellular resistance/antiviral defense against DENV, we used siRNA silencing targeted against each of the 12 homologs in an *Ae. aegypti* cell line (Aag2) infected with DENV2 and identified that silencing of the two candidates, *AeFKBP1* and *AeATCAY*, homologs of human *FKBP1B* and *ATCAY*, were associated with a viral increase. We then used dsRNA to silence each of the two genes in adult mosquitoes to validate the observed antiviral functions in vivo. Depletion of *AeFKBP1* or *AeATCAY* increased viral dissemination through the mosquito at 14 days post-infection. Our results demonstrated that *AeFKBP1* and *AeATCAY* mediate resistance to DENV akin to what has been described for their homologs in humans. *AeFKBP1* and *AeATCAY* provide a rare opportunity to elucidate a DENV-resistance mechanism that may be evolutionarily conserved between humans and *Ae. aegypti*.

## 1. Introduction

Dengue virus (DENV; *Flaviviridae*, Flavivirus) is a causative agent of dengue and dengue hemorrhagic fever. More than 2.5 billion people are at risk of DENV infection, and an estimated 96 million people per year have infections that manifest clinically [1,2]. Recently, a commercially available vaccine was developed, but its safety profile is questionable, leaving the at-risk population without a key preventative measure [3,4,5,6]. The lack of both an effective vaccine and anti-viral therapies make mosquito control and disease surveillance the primary methods to combat DENV transmission. However, *Aedes aegypti*, the primary vector of DENV, is not easily controlled, and surveillance of DENV has not reduced the spread of outbreaks to date [1]. Therefore, novel and effective control methods are needed to reduce DENV transmission. A promising and relatively newer strategy that prevents pathogen transmission is the genetic control of mosquito-borne diseases utilizing loci expressed in the vector [7,8]. Arbovirus infection of vector tissues, particularly the midgut and salivary glands, is an obligate part of the DENV transmission cycle, and despite recent advances in delineating anti-viral defense mechanisms in the vector, our understanding of the underlying pathways and their interconnectivity (i.e., crosstalk) remains incomplete [9]. Hence, it is necessary to elucidate the genetic basis of mosquito immunity to arboviruses as one possible means of developing a more effective dengue control strategy.

Although the process of DENV replication within human and mosquito cells is similar, the virus experiences distinctly different physiologies in each host. In the mosquito, the usual route of infection is oral, and virus particles must therefore infect midgut epithelial cells to perpetuate the transmission cycle. The time interval between oral infection of virus and ability of transmission by bite is termed as the extrinsic incubation period (EIP). The EIP of DENV in *Aedes aegypti* varies based on environmental factors such as temperature or larval nutrition (which affects adult mosquito robustness), but the range is between 7–14 days in controlled studies [10,11,12]. After entering midgut cells through endocytosis, the DENV positive-sense ssRNA genomes are released into the cytoplasm where they get translated into polyproteins that are cleaved into structural and non-structural proteins [13]. Replication of DENV genomes occurs in the cytoplasm within virus-induced, vesicular membranous structures associated with the endoplasmic reticulum (ER) [14]. Newly synthesized RNA genomes associate with capsid proteins and then bud into the ER forming immature virions [15]. These particles mature during transport through the trans-Golgi network and are then released into the mosquito’s open circulatory system either by exocytosis through the basal lamina [16] or by way of the tracheal system [10] or muscle tissue surrounding the midgut [17]. Once in the hemocoel, the flow of hemolymph promotes dissemination of virions to other tissues where the process of infection, replication, and escape is repeated. This amplifies virus production systemically, increasing the likelihood of salivary-gland infection and the production of infectious virions in saliva [10,16,18]. While in mosquito cells, DENV particles and/or replication trigger antiviral pathways, including those responsible for RNA interference and innate immunity. It is now recognized that RNA interference (RNAi) pathways [19,20], particularly the one mediated by exogenous small interfering RNAs (exo-siRNAs), represent the primary antiviral response against DENV [21]. Other RNAi pathways include those mediated by microRNAs (miRNAs) and PIWI (P-element-induced wimpy testes)-interacting RNAs (piRNAs), which primarily regulate endogenous gene expression and transposons, respectively, but have been recently implicated in defense against DENV [22,23,24,25]. Among innate immunity pathways, the Toll [26,27], Janus kinase (JAK)-signal transducer and activator of transcription (STAT) [28,29], and Immune Deficiency (Imd) [30,31] pathways have been shown to elicit antiviral responses in mosquitoes, although the effector mechanisms remain poorly understood. In addition, a number of putative or confirmed arboviral restriction factors have been identified, including DVRF1 (Q1HR00) and DVRF2 (AAEL000896) [28], a putative cystatin (AAEL013287), a hypothetical protein with ankyrin repeats (AAEL003728) [32], Lysozyme C and Cecropin G (AAEL015404) [33], Cecropin (AAEL015515) [31], AeTEP-1 (AAEL012267) [34], and Ubi3881 (AAEL003881) [35]. Considering that much of the study of mosquito immunity has been based on *Drosophila* models [36,37,38], our knowledge of specific DENV resistance genes that may or may not be related to RNAi pathways or the classic innate immune pathways in *Ae. aegypti* is poor. Moreover, since DENV does not naturally infect *Drosophila*, this model has limitations in its complete translation to mosquito-DENV research.

Although our understanding of DENV-mosquito interactions is incomplete, the interaction between DENV and human host cells and tissues has been studied extensively [39]. A genome-wide screen of host-enabling factors and restriction factors to West Nile virus (WNV) or DENV2 in HeLa cells identified 22 WNV-resistance genes that resulted in higher WNV titers following gene silencing. Importantly, these 22 WNV resistance genes were also shown to be involved in resistance to DENV2 infection [40]; suggesting a common resistance mechanism against Flavivirus infections in humans. Here, we explored the possibility that mosquitoes and humans share resistance mechanisms against flaviviruses that supplement the better-characterized viral defense strategies in mosquitoes. To this end, we conducted sequence homology analysis of the 22 human WNV/DENV-resistance genes in the *Ae. aegypti* genome and found 12 *Ae. aegypti* homologs. Functional conservation of these homologs was tested by siRNA silencing using an *Ae. aegypti* cell line, Aag2, and adult mosquitoes. We found that two homologs of the 22 human restriction factors have an anti-DENV function in mosquitoes, suggesting, remarkably, that these proteins have maintained an antiviral role in eukaryotic lineages that diverged approximately 600 million years ago [41,42].

## 2. Materials and Methods

### 2.1. In Silico Identification of Mosquito Homologs for Dengue Virus Resistance (DVR) Homologs

The non-redundant (nr) *Ae. aegypti* genome (VectorBase, VB-2018-10) was searched using TBLASTX for putative homologs of the 22 human WNV/DENV-resistance genes reported by Krishnan et al. [40]. For each query, an E-value of 1 × 10^−20^ was imposed as a cutoff. For queries returning more than one locus meeting this criterion, we selected the one with the best total score as the putative homolog. Of the 22 human genes queried, 12 loci in the *Ae. aegypti* genome were selected for follow-up study.

### 2.2. Mosquito Rearing, Cell Lines, and Viruses

*Aedes aegypti* (Rockefeller/UGAL [Rock]) mosquitoes were maintained on a 10% sucrose solution at 27 °C and 80% humidity with a 12 h light/dark cycle. Dengue virus 2 (DENV; New Guinea C strain, type 2) was cultured and titered according to standard methodology [43]. DENV2 was grown in Vero cells (African green monkey; purchased from ATCC.org, Manassas, VA, USA) once, and the medium was collected and centrifuged at 1000× *g* for 10 min at 4 °C. The resulting supernatant was stored at −80 °C until use. All Vero cells were grown in Dulbecco’s Modified Eagle Medium (DMEM, ThermoFisher, Waltham, MA, USA) with 10% fetal bovine serum (FBS; ThermoFisher), 100 U/mL penicillin, and 100 µg/mL streptomycin (Penicillin-Streptomycin; ThermoFisher) at 37 °C with 5% CO_2_. Aag2 cells (*Aedes aegypti*) were kindly provided by D. O’Brochta (University of Maryland Biotechnology Institute, Rockville, MD, USA) and cultured as originally described by Lan and Fallon [44] with minor modifications. Briefly, Aag2 cells were grown in Leibovitz L15 medium (ThermoFisher) supplemented with 10% tryptose phosphate broth (MP Biomedicals, Santa Ana, CA, USA), 10% FBS, 100 U/mL penicillin, and 100 µg/mL streptomycin at 27 °C without CO_2_.

### 2.3. siRNA Preparation

Target sites for siRNA of the 12 identified *Ae. aegypti* West Nile/dengue virus resistance homologs and the β-gal control were determined using Ambion siRNA Target Finder (ThermoFisher) [45,46] (Table S1). The specificity of each of the 12 siRNAs was predicted in the non-redundant (nr) *Ae. aegypti* genome using the BLASTN algorithm. Sequences were eliminated and re-designed if the target sequences were homologous to other non-targeted coding sequences with more than 15 contiguous base pairs. Synthesis of siRNAs targeting the respective mRNAs was performed commercially using the sequences listed in Table 1 by the Bioneer Company (Daejeon, Korea).

### 2.4. siRNA Transfection

Transfection of siRNAs in Aag2 cells was performed in 24-well plates using Lipofectamine at a final concentration of 200 nM according to the manufacturer’s instructions (ThermoFisher) as previously described [47]. Briefly, when the Aag2 cells reached ~80% confluency, the culture medium was removed and 200 μL of fresh L15 medium without antibiotics and FBS was added. The cells were then transfected with each siRNA in six replicates. After 6 h of incubation at room temperature, transfection medium was replaced with complete medium including proper antibiotics and 10% FBS as described earlier.

### 2.5. Quantitative Real-Time PCR Analysis (qRT-PCR) for Aag2 Cells

Silencing efficiencies of siRNA were estimated using Aag2 cells harvested 3 days after gene silencing through transfection (n = 3 replicates) (Table S2 for primers). Total RNA was isolated from the cells to assess the levels of transcript reduction by two-step qRT-PCR using Trizol Reagent (ThermoFisher). The quality and quantity of RNAs were measured by a NanoDrop spectrophotometer (ThermoFisher). First-strand cDNAs were then synthesized using a SuperScript III first-strand synthesis supermix according to the manufacturer’s instructions (ThermoFisher). Briefly, 500 ng of total RNA was incubated with random hexamers at 65 °C for 5 min to denature the RNAs and subsequently placed on ice. Superscript III with RNaseOUT enzyme mix was then added to the RNA samples. Samples were first incubated at 25 °C for 10 min. The reverse transcription reactions were performed at 50 °C for 50 min followed by inactivation at 85 °C for 5 min. All qPCR reactions and melting curve analyses were performed in triplicate with an IQ5 I-cycler (BioRad, Hercules, CA, USA) in 25 μL reactions containing 12.5 μL SYBR Green PCR Master Mix (BioRad), 0.5 μL of each primer (10 μM), 1.0 μL of cDNA template, and 10.5 μL of ultrapure water. Reactions were carried out at 95 °C for 3 min, followed by 50 cycles of denaturation at 95 °C for 15 s, annealing and extension at 60 °C for 1 min. PCR data were normalized to the Ribosomal Protein S7 (RpS7) gene as an internal control using a mathematical model developed for relative quantification of real-time PCR amplicons [48]. RpS7 has been used to normalize transcript expression levels in *Aedes* mosquito infection studies [30,49,50], as its expression is relatively stable except for a significant decrease 48 h post-blood feeding, after which it returns to levels observed 24 h earlier [51,52].

### 2.6. Virus Infection

To test if the target genes are involved with antiviral defense mechanism, transfected Aag2 cells were infected with DENV2 at 6 h after transfection at a multiplicity of infection (MOI) of 0.05 in 6 replicates. Cell culture media containing DENV2 was subjected to plaque assays in Vero cells at 3 days post-infection (dpi) to assess titers.

### 2.7. Plaque Assay

DENV2 titers in the medium of Aag2 cells transfected with siRNAs were determined by plaque assay [53]. Vero cells were seeded at a density of 2 × 10^5^ cells in each well of a 12-well plate 24 h prior to infection. Ten-fold serial dilutions of the media from each sample were made in DMEM, and 100 μL of each dilution were added to each well. The virus was allowed to infect the cells for 1 h at 37 °C, after which the cells were overlaid with 0.5% agarose in MEM with 10% FBS (Invitrogen, CA). The cells were then incubated at 37 °C with 5% CO_2_ for 3 days, after which a second overlay of agarose was added to each well. The second overlay had the same composition as the first except that it contained 3.3% (v/v) of a 1:300 solution of neutral red (Sigma-Aldrich, St. Louis, MO, USA). Plaques were counted 24 h after the second overlay.

### 2.8. Sequence and Phylogenetic Analyses

Pairwise sequence comparisons of human proteins and *Ae. aegypti* proteins were performed using the BLASTP algorithm with the human FKBP1B (NP_004107) or ATCAY (NP_149053) as queries. When searching FKBP1B against the *Ae. aegypti* genome, six homologs were discovered, but the gene denoted AAEL007833 in VectorBase had the highest score, best alignment length, highest percent amino acid identity (19.5% greater than the next best homolog), and lowest E value. We then performed a reciprocal search by querying AAEL007833 against the human genome and found that the best BLASTP hit was FKBP1A with the next best protein being FKBP1B. So although the human genome codes for two FKBP1 proteins, the *Ae. aegypti* genome appears to have only one, as the other FKBP paralogs have greater similarity to other human FKBP proteins. Therefore, we named the AAEL007883 gene *AeFKBP1*. Phylogenetic analyses were performed using MEGA 5 [54] and a bootstrap consensus tree was obtained using the neighbor-joining method with 1000 bootstrap replications. To construct a phylogenetic tree of FKBP1B homologs, the relevant sequences of human (NP_004107), chimpanzee (*Pan troglodyte*, XP_001144201), cow (*Bos taurus*, NP_001091627), chicken (*Gallus gallus*, NP_989898), zebrafish (*Danio rerio*, NP_957106), mouse (*Mus musculus*, NP_058559), fruit fly (*Drosophila melanogaster*, NP_523792), African malaria mosquito (*An. gambiae*, XP_320351), yellow fever mosquito (*Ae. aegypti*, XP_001652968), red flour beetle (*Tribolium castaneum*, XP_969563), parasitoid wasp (*Nasonia vitripennis*, XP_001599935), honey bee (*Apis mellifera*, XP_624498), silk worm (*Bombyx mori* NP_001040382), black-legged tick (*I. scalpularis*, XP_002406730), and baker’s yeast (*S. cerevisiae*, NP_014264) were obtained from the NCBI sequence database using precalculated HomoloGene (https://www.ncbi.nlm.nih.gov/homologene/?term=homologene) and individual sequence retrieval using the BLASTP algorithm with the human FKBP1B (NP_004107) as a query. The conserved domains of human and mosquito proteins were compared using the DELTA-BLAST (Domain Enhanced Lookup Time Accelerated BLAST) algorithm.

In contrast to FKBP1B, the human ATCAY protein (NP_149053) appears to have four putative homologs in the *Ae. aegypti* genome, all of which are less similar at the amino acid level than FKBP1B/AAEL007833. The best BLASTP hit for ATCAY was a putative cdc42 rho GTPase-activating protein (AAEL021611), and a reciprocal search of the human genome returned numerous proteins (including ATCAY) with very similar scores and percent amino acid identities. For this study, we refer to AAEL021611 as AeATCAY but make no claims about orthology for either pair of human-mosquito homologs.

### 2.9. dsRNA Preparation for Adult Mosquito Experiments

Total RNA was extracted from whole mosquito bodies and cDNA was synthesized with random hexamers. To generate templates for dsRNA synthesis, specific primer sets were designed for each gene of interest (Table S3) and used for PCR with T7 sequences (5’-TAA TAC GAC TCA CTA TAG GG-3’) appended to the 5’ ends. The PCR products were utilized for dsRNA synthesis using the Megascript RNAi Kit (Ambion, CA) following the manufacturer’s instructions.

### 2.10. Gene Silencing Assay in Adult Mosquitoes

RNAi-meditated gene silencing in *Ae. aegypti* mosquitoes was performed to determine if AAEL021611 (AeATCAY) and AAEL007883 (AeFKBP1) have antiviral functions in adult mosquitoes using double-stranded green fluorescence protein (dsGFP) RNA as a negative control. In two groups of 50 mosquitoes per treatment, female mosquitoes three to four-days old were injected with 69 nL of dsRNA (200 ng/mosquito) into the thorax of each mosquito using a nanoinjector (Nanoject II; Drummond Scientific, Broomall, PA, USA) with glass capillary needles. Mosquitoes were maintained in 8 oz paperboard “mosquito cups” covered with mesh and supplied with 20% sucrose to enhance recovery. Three days after injection, mosquitoes were fed blood supplemented with DENV2 using an artificial feeding apparatus (Hemotek, United Kingdom) following previously established methods [55,56,57] or were sacrificed to assess the efficiency of gene silencing. To determine the efficiency of gene silencing, RNA was extracted from 3–5 whole mosquitoes per biological replicate (n = 3 replicates) at three days after injection and analyzed by qRT-PCR. For mosquitoes fed with DENV2, engorged females were maintained in mosquito cups at 28 °C with 20% sucrose solution for 7 days or 14 days post-infection (dpi). At 7 dpi for each treatment, mosquitoes in one cup (n = 25–30) were sacrificed to determine the infection rate (i.e., whole body mosquito infectivity), while the remaining mosquitoes in the other cup (n = 23–42) were sacrificed at 14 dpi. From the latter, legs were removed and saved from individual mosquitoes to assay for viral dissemination, while whole bodies without legs were saved to determine mosquito infectivity at 14 dpi. Only if the virus was detected in mosquito bodies were the corresponding legs processed to investigate viral dissemination (n = 19–38). All samples were homogenized using a Tissue Lyser (Qiagen, CA), and RNA was extracted using Trizol Reagent (ThermoFisher) following previously established methods [56,57]. The amount of DENV2 RNA in the bodies and legs was estimated using qRT-PCR with DENV2-specific primers (Table S4) and the iTaq Universal SYBR Green One-step kit (BioRad) on a Bio-Rad CFX96™ Real-Time PCR Detection System. Mosquito infection rates were presented as the percentage of blood engorged mosquitoes with bodies testing positive for DENV2 RNA at 7 and 14 dpi. Dissemination rates were calculated as the percentage of mosquitoes with DENV2-positive bodies that also had DENV2-positive legs at 14 dpi as described elsewhere [58].

### 2.11. Gene Expression during Infection

Approximately 50 female mosquitoes were fed with infectious or naïve blood. Whole bodies of three to five groups of three mosquitoes were homogenized at 0 (1 h after blood feeding), 7, and 14 days after blood feeding. Total RNA was extracted using Trizol Reagent (ThermoFisher), and cDNA was prepared using a SuperScript III first-strand synthesis supermix according to the manufacturer’s instructions (ThermoFisher). Induction of AeFKBP1 and AeATCAY during infection was quantified using qRT-PCR with gene specific primers as described above in the sub-section on qRT-PCR for Aag2 cells. The exon sequences of each gene were exported from the *Ae. aegypti* complete transcript database (VectorBase, VB-2018-10). The primer sets were designed based on the exon sequences, using the IDT PrimerQuest Tool (Integrated DNA Technologies, Skokie, IL, USA)). The ribosomal protein gene S7 was used as an endogenous control (Table S4).

### 2.12. Statistical Analysis

Statistical analyses were performed by the Mann-Whitney U test or two-way analysis of variance (ANOVA) using the GraphPad Prism software package (Prism 5.05; GraphPad Software, Inc., San Diego, CA, USA).

## 3. Results

### 3.1. Silencing of Two Aedes aegypti Homologs of WNV/DENV2-Resistance Genes Increased DENV2 Infection in Aag2 Cells

**Figure 1:**
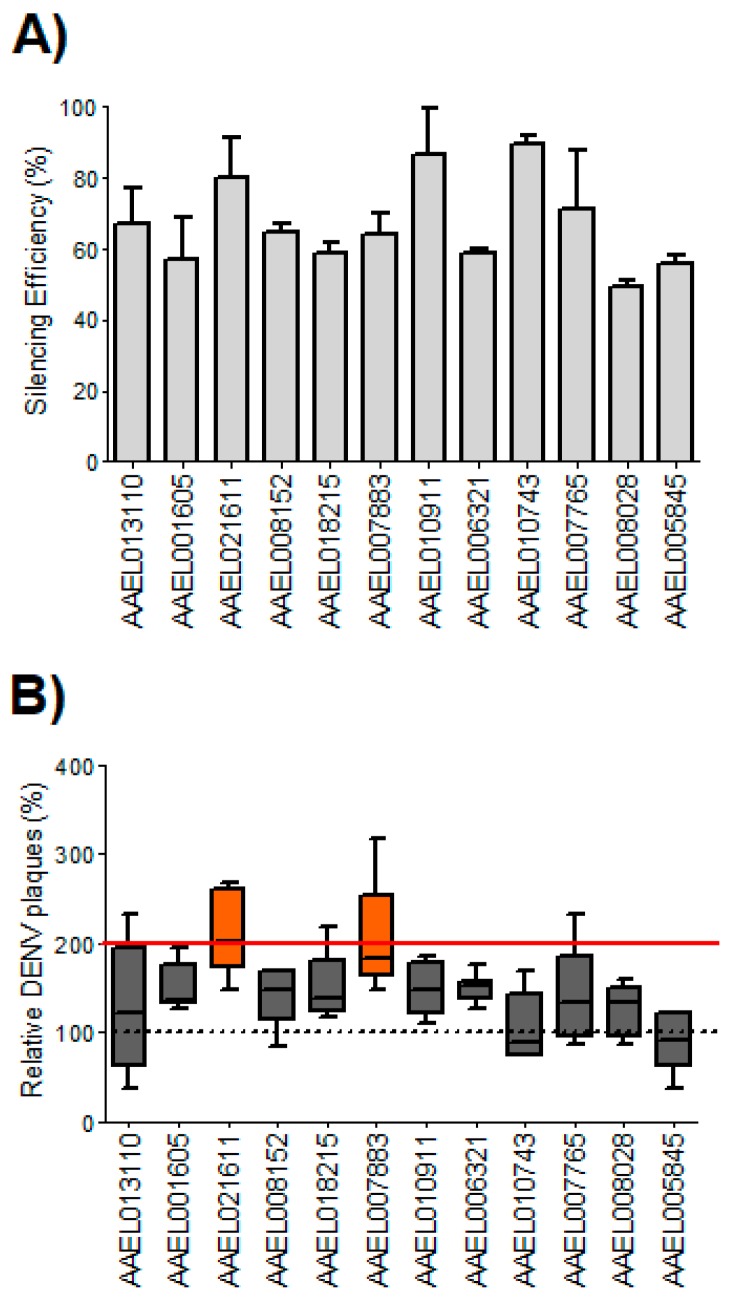
siRNA-mediated silencing assay during Dengue virus 2 (DENV2) infection in Aag2 cells. (**A**) Silencing efficiencies were determined by quantitative real-time PCR compared to the non-silenced control (β-gal siRNA transfected) and presented as means and standard errors (n = 3). Mean silencing efficiencies ranged from 49.37% to 89.34% compared to the control group. (**B**) Putative anti-DENV2 function of twelve homologs of human West Nile virus (WNV)/DENV2 resistance genes were tested using siRNA silencing assays in Aag2 cells. For each assay result, DENV2 titers are presented as means and standard errors (n = 6). The highlighted bars (AAEL021611 and AAEL007883;) indicate that silencing of the genes increased DENV2 titers (Mann-Whitney U test, *p* = 0.002 and *p* = 0.008, respectively) nearly two-fold higher than the β-gal silenced controls (normalized to 100%, dotted line). The red line indicates a two-fold increase over controls.

We identified 12 *Ae. aegypti* homologs out of 22 human WNV/DENV2-resistance genes [40] (Table 1) and explored their putative roles in DENV2 infection in mosquitoes. We silenced expression of all 12 genes using RNA interference in Aag2 cells and examined the impact on DENV2 infection. To confirm gene silencing and assess efficiency of knock-down prior to infection studies, RNA was isolated from the transfected cells at Day 3 and used in qPCR analysis to measure gene expression (Figure 1A). Compared to the non-silenced control (β-gal siRNA transfected), mean silencing efficiencies ranged from 49.37% to 89.34%. To quantify the effects of siRNA silencing of target mRNAs on DENV2 titers, the culture media of DENV2-infected Aag2 cells (MOI = 0.05) that had been silenced for each target mRNA were collected at 3 dpi and used in plaque assays to calculate viral titers. Viral titers were normalized to the control experiment (β-gal siRNA) defined as 1. The average viral titer was higher than the control in 10 groups out of 12, but silencing of two genes (AAEL021611, *AeATCAY*; and AAEL007883, *AeFKBP1*) increased viral titers significantly (*p* = 0.002 and *p* = 0.008, respectively), reaching titers approximately two-fold higher than controls (Figure 1B and Table S5). Given that a two-fold increase in viral titer has been considered a meaningful, biologically relevant increase in mosquito functional genomics studies that used an RNAi approach [30,49,59], we selected the *AeATCAY* and *AeFKBP1* gene targets for in vivo validation.

### 3.2. Sequence Analyses Indicate a High Degree of Domain Conservation for FKBP1 and ATCAY between Humans and Mosquitoes

**Figure 2:**
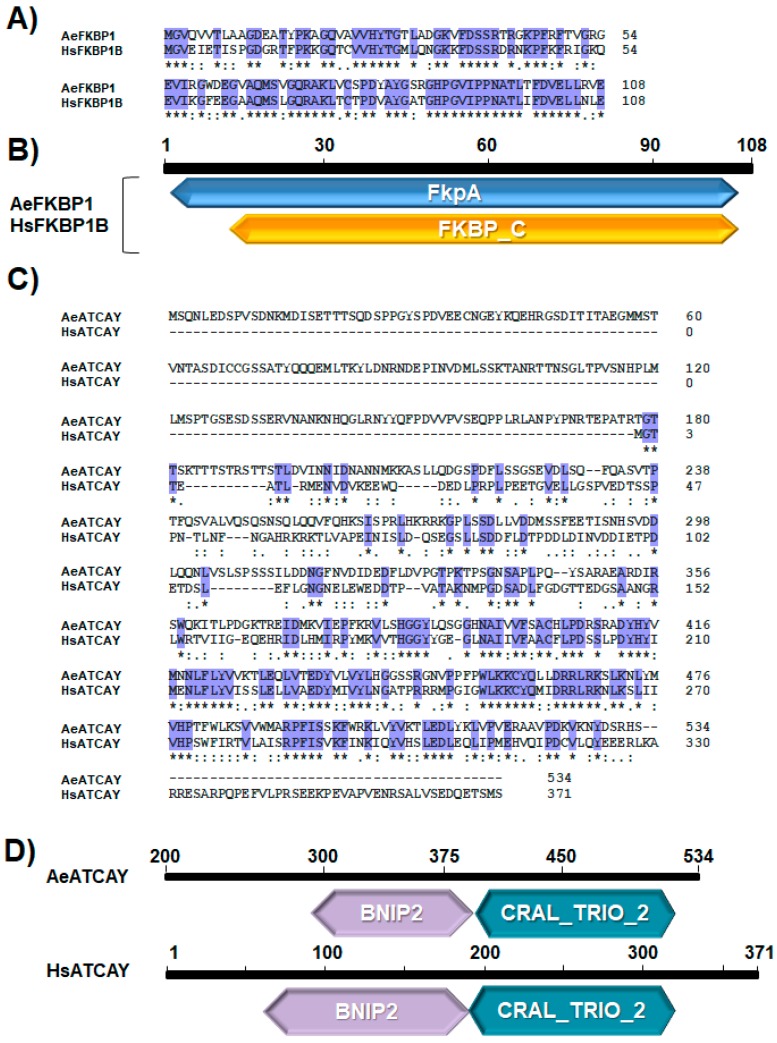
Sequence analysis of AeFKBP1 and AeATCAY. (**A**) Pairwise sequence alignment of human FKBP1B and AeFKBP1 shows that both sequences share 72.2% and 81% amino acid identity and similarity, respectively. These two proteins consist of 108 amino acids without gaps, indicating putative functional conservation. (**B**) Comparison of protein domains using the DELTA-BLAST algorithm shows that human FKBP1B (HsFKBP1B) and AeFKBP1 share FKBP-type peptidyl-prolyl cis-trans isomerase domains. (**C**) Pairwise sequence alignment of human ATCAY (HsATCAY) and AeATCAY shows that both sequences share 48% and 69% amino acid identity and similarity, respectively. (**D**) Comparison of protein domains using the DELTA-BLAST algorithm shows that human ATCAY and AeATCAY share BNIP2 and CRAL/TRIO domains but differ in in domain length. (**A**,**C**) ‘*’ means fully conserved residue; ‘:’ means strongly similar properties; ‘.’ means weakly similar properties.

A pairwise sequence alignment of human FKBP1B and AeFKBP1 showed that they share 72.2% and 81% amino acid identity and similarity, respectively, and maintain the same protein length (108 amino acids; no gaps) (Figure 2A). Therefore, the human FKBP1B (HsFKBP1B) and AeFKBP1 appear to be homologous proteins with a highly conserved primary structure, suggesting the potential for functional conservation. Both proteins share FKBP-type peptidylproline cis-trans isomerase (PPIase) domains, which are found in proteins collectively called immunophilins that act as co-regulatory subunits of molecular complexes involved in the folding, assembly, and trafficking of proteins, as well as immunomodulation and apoptosis [60,61] (Figure 2B). In vertebrates, FKBP-type immunophilins are expressed in a wide range of cell types, and isoforms may be secreted or membrane-bound [60,61]. The human protein FKBP1A, a paralog of FKBP1B, is known to bind to the immunosuppressants FK506 (*K_d_* = 0.4 nM) and rapamycin (*K_d_* = 0.2 nM) and has been implicated in T-cell activation and proliferation [62]. Phylogenetic analysis revealed that FKBP1 homologs share considerable sequence conservation among eukaryotes from yeast to humans (Figure S1). Even when human FKBP1B was compared with the most distant taxon, *Saccharomyces cerevisiae*, the amino acid identity and similarity were 56% and 74%, respectively. This high degree of sequence conservation across widely diverse taxa indicates fundamental importance of FKBP1 proteins in cellular processes, likely stemming from their small size and the multifunctional nature of FKBP-type immunophilins [61].

We also compared ATCAY from humans (HsATCAY) and *Ae*. *aegypti* and found that they share 48% and 69% amino acid identity and similarity, respectively (Figure 2C). Compared to FKBP1, the percent identity between mosquito and human homologs is not as high; however, both proteins contain the BNIP2 and CRAL/TRIO domains (Figure 2D). BNIP2 domains interact with apoptosis regulators, such as BCL2 [63,64], and CRAL/TRIO domains have been known to suppress Newcastle disease virus in a human cell line [65], suggesting a potential mechanism of anti-DENV function of this gene in mosquitoes.

### 3.3. Viral Dissemination in Adult Mosquitoes was Increased Following the Silencing of AeATCAY and AeFKBP1 at 14 dpi

**Figure 3:**
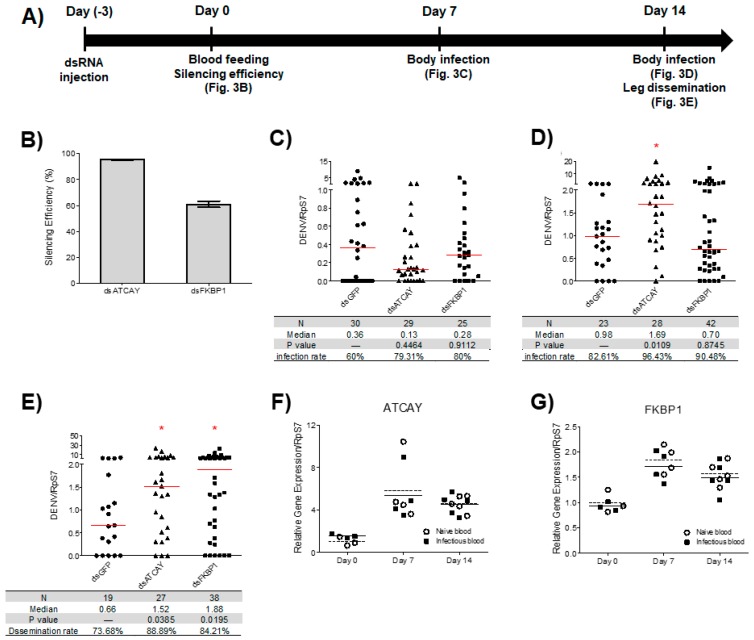
dsRNA-mediated silencing assay during DENV2 infection in adult mosquitoes. Mosquitoes were injected with dsRNA against AeATCAY (AAEL021611) or AeFKBP1 (AAEL007883) and infected with DENV2 through infectious blood feeding. (**A**) Experimental design. (**B**) Silencing efficiencies were determined by quantitative real-time PCR compared to the control (dsGFP injected) and presented as means (60.92% for AeFKBP1 and 95.47% for Ae ATCAY) and standard errors (n = 3). (**C**) Relative viral RNA was measured in whole bodies at 7 days post-infection (dpi) and compared to the control. (**D**,**E**) Relative viral RNA was measured in whole bodies without legs (**D**) and in legs (**E**) at 14 dpi. (**C**–**E**) DENV2 RNA copy number was normalized to RpS7 (AAEL009496) and compared to the control (dsGFP injected). The red bar indicates a relative and normalized median DENV2 copy number. Statistical analyses were performed by the Mann-Whitney U test. Gene expression of (**F**) AeATCAY (AAEL021611) and (**G**) AeFKBP1 (AAEL007883) in infected mosquitoes were compared to naïve blood-fed mosquitoes at 0, 7 and 14 days post-infection. Transcript levels of both genes were not affected by DENV2 infection in mosquitoes at 7 and 14 days post-blood feeding (**F**,**G**) Statistical analyses were performed by two way ANOVA; (*F*(1, 18) = 0.0001322, *p* = 0.9910) for AeATCAY; (*F*_(1, 18)_ = 0.7567, *p* = 0.3958) for AeFKBP1.

We investigated whether silencing the two genes that significantly influenced DENV2 titers in Aag2 cells (AeATCAY and AeFKBP1) would impact virus titers in adult mosquitoes. We injected mosquitoes with dsRNA against these genes three days prior to feeding on DENV2-mixed with blood (Figure 3A). To confirm gene silencing, RNA was isolated from a subset of whole mosquitoes at three days after dsRNA injection and used in qRT-PCR analysis to measure target gene expression (Figure 3B). To examine DENV2 RNA levels in mosquitoes, relative amounts of DENV2 RNA were normalized to the housekeeping gene RpS7, which has been used to measure amount of infected viruses in mosquitoes [66]. Silencing the genes did not affect relative DENV2 RNA in the total mosquito body at 7 dpi (Figure 3C).

To analyze the impact of gene silencing on DENV2 dissemination, which is calculated as the percentage of mosquitoes at 14 dpi with DENV2-positive bodies that also had DENV2-positive legs, we looked at relative DENV2 RNA at day 14 dpi. Silencing AeATCAY increased relative DENV2 RNA levels by 1.7-fold (*p* = 0.0109) in mosquito bodies compared to controls (Figure 3D), while silencing AeFKBP1 did not have an effect (Figure 3D). To analyze dissemination rate, DENV2 RNA was quantified in RNA extracted from mosquito legs at day 14 dpi. Silencing AeATCAY and AeFKBP1 significantly increased relative DENV2 RNA in legs by 2.3- and 2.8-fold, respectively (*p* = 0.0385 and *p* = 0.0195) (Figure 3E).

### 3.4. Expression of Selected DENV2 Resistance Genes Increased during Blood Feeding But Was Unaffected by Viral Infection

To determine if DENV2 infection influences the expression of these putative resistance genes, we quantified gene expression during infection at various time points. We analyzed transcriptional changes in the genes in whole mosquitoes either fed with DENV2 infectious blood or naïve blood. RNA was isolated at 0 (1 h after feeding), 7, and 14 days post-blood feeding (dpf), and gene expression was quantified by qPCR. Our results showed that expression of AeATCAY, in both infected and uninfected samples, increased by ~1.8-fold at 7 dpf and by ~1.5-fold at 14 dpf compared to the day of feeding (*F*_(2, 18)_ = 11.21, *p* = 0.0007) (Figure 3F). The pattern was similar for the expression of AeFKBP1, although the relative increase observed was greater. AeFKBP1 expression of both infected and uninfected samples increased during blood feeding, by ~5.8-fold at 7 dpf and ~4.5-fold at 14 dpf (*F*_(2, 18)_ = 17.77, *p* < 0.0001) (Figure 3G). However, our data indicated that the transcript levels of the two genes were unaffected by DENV2 infection. In other words, transcript levels were similar at 7 and 14 dpf with infectious or naïve blood ((*F*_(1, 18)_ = 0.0001322, *p* = 0.9910) for AeATCAY; (*F*_(1, 18)_ = 0.7567, *p* = 0.3958) for AeFKBP1).

## 4. Discussion

We identified 12 *Ae. aegypti* homologs of 22 previously reported human WNV/DENV2-resistance genes [40]. We cannot rule out the possibility that other mosquito homologs of these genes have anti-DENV function, but as a first step, we focused on the 12 genes with the lowest E values and best BLAST scores. Considering functional similarities of some of the homologs involved in innate immunity [67], we hypothesized that some of these 12 might be functionally conserved between human and *Ae. aegypti* with respect to imparting DENV2-resistance, particularly since both organisms are part of the natural transmission cycle. As obligate intracellular pathogens, arboviruses alternate between vertebrate and arthropod hosts and require ‘host factors’ to complete their life cycle in each organism, as they lack most of the cellular machinery necessary for replication [68]. Because determinants of infection status include host antiviral defense, in addition to these host replication factors, a better understanding of mosquito-DENV2 interactions is a prerequisite to the discovery of target molecules that may be exploited for more effective approaches to interrupt DENV2 transmission [69]. Our siRNA-based knockdown assays of the 12 *Ae. aegypti* homologs revealed that *AeFKBP1* and *AeATCAY* may encode DENV2 resistance factors much like human *FKBP1B* and *ATCAY* genes. Therefore, we can infer that a common cellular defense mechanism mediated through these two genes may be conserved in both *Ae. aegypti* and humans. We further validated the in vitro assay by in vivo RNAi knockdown in adult mosquitoes. Interestingly, silencing of *AeFKBP1* and *AeATCAY* did not increase viral titers in the whole body at 7 dpi. Our results suggest that neither gene acts as a restriction factor for viral infection or replication in the midgut (i.e., not a component of the midgut infection barrier), or at least not until after 7 dpi.

The lack of congruence between the impact of silencing each gene on whole-body and disseminated infections at day 14 (Figure 3D,E, respectively) may be because the influence of each gene on viral infection and/or dissemination is different. Comparison of Figure 3D,E shows that at day 14 post-infection, silencing AeATCAY increased viral copy numbers in both whole body and legs while silencing AeFKBP1 increased the amount of virus only in legs (i.e., a tissue that marks disseminated infection). Figure 3D illustrates potential roles of target genes in virus propagation but does not provide data relevant for systemic viral dissemination (i.e., processes that occur following escape from the midgut epithelium). To investigate dissemination, we measured viral RNA using only legs. Figure 3E shows that at day 14 post-infection, viral dissemination is reduced compared to controls, implying that the two genes influence the process of dissemination. Therefore, it appears that AeFKBP1 may be associated with a barrier that slows the pace of DENV2 dissemination but may not affect viral propagation. In contrast, AeATCAY appears to suppress viral propagation and dissemination between 7 dpi and 14 dpi. Compared to controls, more viral RNA was detected in the AeATCAY-silenced samples for both whole body and legs at 14 dpi, suggesting that AeATCAY may be involved in suppression of viral propagation and dissemination after the virus passes the midgut barrier.

In a relevant study, Behura et al. reported differential modulation of gene expression between DENV-refractory (Moyo-In-Dry) and susceptible (DS3) *Ae. aegypti* strains in response to DENV infection [70]. In that experiment, *AeFKBP1* was found to be transcriptionally up-regulated by about five-fold in the midgut of the refractory strain when compared to the susceptible strain at 3 days post DENV2 infection. As for the response of *AeATCAY* gene expression to DENV infection, there are at least two contradictory studies. Colpitts et al. did not observe any statistically significant changes in expression of *AeATCAY* at 1, 2, and 7 dpi with DENV2 when the virus was intrathoracically injected [52], while Behura et al. reported that *AeATCAY* is up-regulated in a refractory strain and down-regulated in a susceptible strain early in a DENV2 infection [71]. Therefore, expression of *AeFKBP1* and *AeATCAY* may be differentially regulated in a tissue-specific or strain-specific manner. However, in our study we found that both *AeFKBP1* and *AeATCAY* expression levels remained unaffected by DENV2 infection, albeit our experimental design did not include time points earlier than 7 dpi precluding direct comparisons with data from the two studies by Behura and colleagues.

We note that the antiviral effects of *AeFKBP1* and *AeATCAY* are less pronounced than those of other known viral defense genes in mosquitoes, such as *ago2* and *dcr2* [21]. However, these genes may share functional conservation with their human homologs, while the siRNA-mediated pathway for viral defense occurs in arthropods but not mammals. The mechanisms underlying the antiviral effects of *AeFKBP1* and *AeATCAY* in mosquitoes remain to be determined, but it is intriguing that these genes may represent remnants of evolutionarily ancient viral-defense strategies. Interestingly, FKBP proteins have been associated with apoptosis regulation in mammals [72], a process that serves as a viral defense mechanism in vertebrates [73]. ATCAY may also be linked to apoptotic pathways, as it has a BNIP (BCL2/adenovirus E1B 19 kd-interacting protein) 2 domain, and proteins of the BCL2 family interact with regulatory proteins that influence apoptosis [63,64]. Whether apoptosis is a limiting factor of viral replication in mosquitoes remains controversial (reviewed in [74]), yet it reportedly coincides with viral infection in *Ae. aegypti* [75], *Ae. albopictus* [76] and *Culex spp*. [17,77,78]. Moreover, there is also evidence that apoptosis limits viral infection in *Drosophila melanogaster* [79,80]. A next step will be to test the hypothesis that the antiviral functions of *AeFKBP1* and *AeATCAY* are exerted through apoptosis.

## 5. Conclusions

In conclusion, our data demonstrate that FKBP1 and ATCAY play anti-DENV roles in human cell lines and *Ae. aegypti*, revealing a potentially ancient antiviral defense mechanism. Silencing of *AeFKBP1*, and *AeATCAY*, homologs of human *FKBP1B* and *ATCAY*, both in an *Ae. aegypti* cell line and adult mosquitoes increases DENV2 viral titers. Human FKBP1B and AeFKBP1 have substantial conservation in their primary structures, and FKBP sequences are highly conserved among various organisms. Together, these data suggest that its anti-DENV function may have appeared during the early evolution of antiviral immunity. Intriguingly, both genes are involved in the regulation of apoptosis in mammals, indicating two potential links to apoptosis as an important mechanism that limits DENV infection and dissemination in *Aedes*. Moreover, given that apoptosis is a well-studied process, proteins in certain parts of the pathway are logical candidates for interaction partners of AeFKBP1 or AeATCAY. These could be targeted experimentally to delineate the pathway(s) underlying apoptosis regulation in response to DENV infection. We hypothesize that these FKBP1- and ATCAY-mediated antiviral pathways represent ancient cellular defense mechanisms, but it remains to be seen if and how they are linked to the canonical pathways of innate immunity.

## Figures and Tables

**Table 1:** Twelve *Aedes aegypti* homologs of human West Nile/dengue virus-resistance genes reported by Krishnan et al [40].

Human WNV/DVR Genes *	*Ae. aegypti* Gene ID (VectorBase, VB-2018-10)	E Value
NM_052957.4	Conserved hypothetical protein (AAEL013110)	6e^−35^
NM_006401.2	Putative microtubule binding protein (AAEL001605)	2e^−33^
NM_033064.4	Putative cdc42 rho GTPase-activating protein (AAEL021611)	1e^−49^
NM_016246.2	Short-chain dehydrogenase (AAEL008152)	1e^−26^
NM_014705.3	Dedicator of cytokinesis (AAEL018215)	0.0
NM_004116.4	FK506-binding protein (AAEL007883)	6e^−39^
NM_032505.2	Kelch-like protein diablo (AAEL010911)	4e^−45^
NM_014873.2	1-Acylglycerol-3-phosphate acyltransferase (AAEL006321)	1e^−56^
NM_021100.4	Cysteine desulfurylase (AAEL010743)	0.0
NM_003784.3	Serine protease inhibitor 10 (AAEL007765)	3e^−34^
NM_004696.2	Monocarboxylate transporter (AAEL008028)	3e^−30^
NM_020971.2	Beta chain spectrin (AAEL005845)	0.0

* The remaining West Nile/dengue virus-resistance genes reported by Krishnan et al. [40] such as NM_001143.1, NM_005217.3, NM_024974.2, NM_001571.5, NM_014804.2, NM_002443.3, NM_006985.3, NM_006993.2, NM_002407.2, and NM_152784.3 had no significant BLAST hits in the *Ae. aegypti* genome database.

## Supplementary Materials

The following are available online at http://www.mdpi.com/2075-4450/10/2/46/s1.

## References

[1] Gubler D.J. (1998). Dengue and dengue hemorrhagic fever. Clin. Microbiol. Rev..

[2] Bhatt S., Gething P.W., Brady O.J., Messina J.P., Farlow A.W., Moyes C.L., Drake J.M., Brownstein J.S., Hoen A.G., Sankoh O. (2013). The global distribution and burden of dengue. Nature.

[3] Capeding M.R., Tran N.H., Hadinegoro S.R.S., Ismail H.I.H.J.M., Chotpitayasunondh T., Chua M.N., Luong C.Q., Rusmil K., Wirawan D.N., Nallusamy R. (2014). Clinical efficacy and safety of a novel tetravalent dengue vaccine in healthy children in Asia: A phase 3, randomised, observer-masked, placebo-controlled trial. Lancet.

[4] Villar L., Dayan G.H., Arredondo-García J.L., Rivera D.M., Cunha R., Deseda C., Reynales H., Costa M.S., Morales-Ramírez J.O., Carrasquilla G. (2015). Efficacy of a tetravalent dengue vaccine in children in Latin America. N. Engl. J. Med..

[5] Sabchareon A., Wallace D., Sirivichayakul C., Limkittikul K., Chanthavanich P., Suvannadabba S., Jiwariyavej V., Dulyachai W., Pengsaa K., Wartel T.A. (2012). Protective efficacy of the recombinant, live-attenuated, CYD tetravalent dengue vaccine in Thai schoolchildren: A randomised, controlled phase 2b trial. Lancet.

[6] Ferguson N.M., Rodríguez-Barraquer I., Dorigatti I., Mier-y-Teran-Romero L., Laydon D.J., Cummings D.A.T. (2016). Benefits and risks of the Sanofi-Pasteur dengue vaccine: Modeling optimal deployment. Science.

[7] Knols B.G., Bossin H.C., Mukabana W.R., Robinson A.S. (2007). Transgenic mosquitoes and the fight against malaria: Managing technology push in a turbulent GMO world. Am. J. Trop. Med. Hyg..

[8] Benelli G., Mehlhorn H. (2016). Declining malaria, rising of dengue and Zika virus: Insights for mosquito vector control. Parasitol. Res..

[9] Cheng G., Liu Y., Wang P., Xiao X. (2016). Mosquito defense strategies against viral infection. Trends Parasitol..

[10] Salazar M.I., Richardson J.H., Sánchez-Vargas I., Olson K.E., Beaty B.J. (2007). Dengue virus type 2: Replication and tropisms in orally infected *Aedes aegypti* mosquitoes. BMC Microbiol..

[11] Black W.C., Bennett K.E., Gorrochótegui-Escalante N., Barillas-Mury C.V., Fernández-Salas I., de Lourdes Muñoz M.A., Farfán-Alé J.A., Olson K.E., Beaty B.J. (2002). Flavivirus Susceptibility in *Aedes aegypti*. Arch. Med Res..

[12] Watts D.M., Burke D.S., Harrison B.A., Whitmire R.E., Nisalak A. (1987). Effect of temperature on the vector efficiency of *Aedes aegypti* for dengue 2 virus. Am. J. Trop. Med. Hyg..

[13] Preugschat F., Strauss J.H. (1991). Processing of nonstructural proteins NS4A and NS4B of dengue 2 virus in vitro and in vivo. Virology.

[14] Jain B., Chaturvedi U.C., Jain A. (2014). Role of intracellular events in the pathogenesis of dengue; An overview. Microb. Pathog..

[15] Byk L.A., Gamarnik A.V. (2016). Properties and functions of the dengue virus capsid protein. Annu. Rev. Virol..

[16] Franz A.W., Kantor A.M., Passarelli A.L., Clem R.J. (2015). Tissue barriers to arbovirus infection in mosquitoes. Viruses.

[17] Girard Y.A., Popov V., Wen J., Han V., Higgs S. (2005). Ultrastructural study of West Nile virus pathogenesis in *Culex pipiens quinquefasciatus* (Diptera: Culicidae). J. Med. Entomol..

[18] Rückert C., Ebel G.D. (2018). How Ddo virus–mosquito interactions lead to viral emergence?. Trends Parasitol..

[19] Travanty E.A., Adelman Z.N., Franz A.W.E., Keene K.M., Beaty B.J., Blair C.D., James A.A., Olson K.E. (2004). Using RNA interference to develop dengue virus resistance in genetically modified *Aedes aegypti*. Insect Biochem. Mol. Biol..

[20] Franz A.W.E., Sanchez-Vargas I., Adelman Z.N., Blair C.D., Beaty B.J., James A.A., Olson K.E. (2006). Engineering RNA interference-based resistance to dengue virus type 2 in genetically modified *Aedes aegypti*. Proc. Natl. Acad. Sci. USA.

[21] Sánchez-Vargas I., Scott J.C., Poole-Smith B.K., Franz A.W.E., Barbosa-Solomieu V., Wilusz J., Olson K.E., Blair C.D. (2009). Dengue virus type 2 infections of *Aedes aegypti* are modulated by the mosquito’s RNA interference pathway. PLoS Pathog..

[22] Yen P.-S., James A., Li J.-C., Chen C.-H., Failloux A.-B. (2018). Synthetic miRNAs induce dual arboviral-resistance phenotypes in the vector mosquito *Aedes aegypti*. Commun. Biol..

[23] Shahen M., Guo Z., Shar A.H., Ebaid R., Tao Q., Zhang W., Wu Z., Bai Y., Fu Y., Zheng C. (2018). Dengue virus causes changes of MicroRNA-genes regulatory network revealing potential targets for antiviral drugs. BMC Syst. Biol..

[24] Wang Y., Jin B., Liu P., Li J., Chen X., Gu J. (2018). piRNA profiling of dengue virus type 2-infected Asian tiger mosquito and midgut tissues. Viruses.

[25] Hess A.M., Prasad A.N., Ptitsyn A., Ebel G.D., Olson K.E., Barbacioru C., Monighetti C., Campbell C.L. (2011). Small RNA profiling of Dengue virus-mosquito interactions implicates the PIWI RNA pathway in anti-viral defense. BMC Microbiol..

[26] Zambon R.A., Nandakumar M., Vakharia V.N., Wu L.P. (2005). The Toll pathway is important for an antiviral response in *Drosophila*. Proc. Natl. Acad. Sci. USA.

[27] Smartt C.T., Shin D., Alto B.W. (2017). Dengue serotype-specific immune response in *Aedes aegypti* and *Aedes albopictus*. Mem. Inst. Oswaldo Cruz.

[28] Souza-Neto J.A., Sim S., Dimopoulos G. (2009). An evolutionary conserved function of the JAK-STAT pathway in anti-dengue defense. Proc. Natl. Acad. Sci. USA.

[29] Jupatanakul N., Sim S., Angleró-Rodríguez Y.I., Souza-Neto J., Das S., Poti K.E., Rossi S.L., Bergren N., Vasilakis N., Dimopoulos G. (2017). Engineered *Aedes aegypti* JAK/STAT pathway-mediated immunity to dengue virus. PLoS Negl. Trop. Dis..

[30] Sim S., Jupatanakul N., Ramirez J.L., Kang S., Romero-Vivas C.M., Mohammed H., Dimopoulos G. (2013). Transcriptomic profiling of diverse *Aedes aegypti* strains reveals increased basal-level immune activation in dengue virus-refractory populations and identifies novel virus-vector molecular interactions. PLoS Negl. Trop. Dis..

[31] Luplertlop N., Surasombatpattana P., Patramool S., Dumas E., Wasinpiyamongkol L., Saune L., Hamel R., Bernard E., Sereno D., Thomas F. (2011). Induction of a peptide with activity against a broad spectrum of pathogens in the *Aedes aegypti* salivary gland, following infection with dengue virus. PLoS Pathog..

[32] Sim S., Ramirez J.L., Dimopoulos G. (2012). Dengue virus infection of the *Aedes aegypti* salivary gland and chemosensory apparatus induces genes that modulate infection and blood-feeding behavior. PLoS Pathog..

[33] Ramirez J.L., Souza-Neto J., Torres Cosme R., Rovira J., Ortiz A., Pascale J.M., Dimopoulos G. (2012). Reciprocal tripartite interactions between the *Aedes aegypti* midgut microbiota, innate immune system and dengue virus influences vector competence. PLoS Negl. Trop. Dis..

[34] Cheng G., Liu L., Wang P., Zhang Y., Zhao Y.O., Colpitts T.M., Feitosa F., Anderson J.F., Fikrig E. (2011). An *in vivo* transfection approach elucidates a role for *Aedes aegypti* thioester-containing proteins in Flaviviral infection. PLoS ONE.

[35] Troupin A., Londono-Renteria B., Conway M.J., Cloherty E., Jameson S., Higgs S., Vanlandingham D.L., Fikrig E., Colpitts T.M. (2016). A novel mosquito ubiquitin targets viral envelope protein for degradation and reduces virion production during dengue virus infection. Biochim. Biophys. Acta.

[36] Sessions O.M., Barrows N.J., Souza-Neto J.A., Robinson T.J., Hershey C.L., Rodgers M.A., Ramirez J.L., Dimopoulos G., Yang P.L., Pearson J.L. (2009). Discovery of insect and human dengue virus host factors. Nature.

[37] Sim S., Jupatanakul N., Dimopoulos G. (2014). Mosquito immunity against arboviruses. Viruses.

[38] Palmer H.W., Varghese S.F., van Rij P.R. (2018). Natural variation in resistance to virus infection in Dipteran Insects. Viruses.

[39] Ngono A.E., Shresta S. (2018). Immune Response to Dengue and Zika. Annu. Rev. Immunol..

[40] Krishnan M.N., Ng A., Sukumaran B., Gilfoy F.D., Uchil P.D., Sultana H., Brass A.L., Adametz R., Tsui M., Qian F. (2008). RNA interference screen for human genes associated with West Nile virus infection. Nature.

[41] Peterson K.J., Cotton J.A., Gehling J.G., Pisani D. (2008). The Ediacaran emergence of bilaterians: Congruence between the genetic and the geological fossil records. Philos. Trans. R. Soc. B Biol. Sci..

[42] Edgecombe G.D., Giribet G., Dunn C.W., Hejnol A., Kristensen R.M., Neves R.C., Rouse G.W., Worsaae K., Sørensen M.V. (2011). Higher-level metazoan relationships: Recent progress and remaining questions. Org. Divers. Evol..

[43] Hierholzer J.C., Killington R.A., Mahy B.W., Kangro H.O. (1996). Virus isolation and quantitation. Virology Methods Manual.

[44] Lan Q., Fallon A.M. (1990). Small heat shock proteins distinguish between two mosquito species and confirm identity of their cell lines. Am. J. Trop. Med. Hyg..

[45] Ganjalikhani Hakemi M., Ghaedi K., Andalib A., Homayouni V., Hosseini M., Rezaei A. (2013). RORC2 gene silencing in human Th17 cells by siRNA: Design and evaluation of highly efficient siRNA. Avicenna J. Med. Biotechnol..

[46] Birmingham A., Anderson E., Sullivan K., Reynolds A., Boese Q., Leake D., Karpilow J., Khvorova A. (2007). A protocol for designing siRNAs with high functionality and specificity. Nat. Protoc..

[47] Kang S., Sim C., Byrd B.D., Collins F.H., Hong Y.S. (2008). Ex vivo promoter analysis of antiviral heat shock cognate 70B gene in *Anopheles gambiae*. Virol. J..

[48] Pfaffl M.W. (2001). A new mathematical model for relative quantification in real-time RT-PCR. Nucleic Acids Res..

[49] Kang S., Shields A.R., Jupatanakul N., Dimopoulos G. (2014). Suppressing dengue-2 infection by chemical inhibition of *Aedes aegypti* host factors. PLoS Negl. Trop. Dis..

[50] Zhang M., Zheng X., Wu Y., Gan M., He A., Li Z., Liu J., Zhan X. (2010). Quantitative analysis of replication and tropisms of Dengue virus type 2 in *Aedes albopictus*. Am. J. Trop. Med. Hyg..

[51] Dissanayake S.N., Ribeiro J.M., Wang M.H., Dunn W.A., Yan G., James A.A., Marinotti O. (2010). aeGEPUCI: A database of gene expression in the dengue vector mosquito, *Aedes aegypti*. BMC Res. Notes.

[52] Colpitts T.M., Cox J., Vanlandingham D.L., Feitosa F.M., Cheng G., Kurscheid S., Wang P., Krishnan M.N., Higgs S., Fikrig E. (2011). Alterations in the *Aedes aegypti* transcriptome during infection with West Nile, dengue and yellow fever viruses. PLoS Pathog..

[53] Schmidt N.J., Dennis J., Lennette E.H. (1976). Plaque reduction neutralization test for human cytomegalovirus based upon enhanced uptake of neutral red by virus-infected cells. J. Clin. Microbiol..

[54] Tamura K., Peterson D., Peterson N., Stecher G., Nei M., Kumar S. (2011). MEGA5: Molecular evolutionary genetics analysis using maximum likelihood, evolutionary distance, and maximum parsimony methods. Mol. Biol. Evol..

[55] Chouin-Carneiro T., Vega-Rua A., Vazeille M., Yebakima A., Girod R., Goindin D., Dupont-Rouzeyrol M., Lourenço-de-Oliveira R., Failloux A.-B. (2016). Differential susceptibilities of *Aedes aegypti* and *Aedes albopictus* from the Americas to Zika virus. PLoS Negl. Trop. Dis..

[56] Shin D., Richards S.L., Alto B.W., Bettinardi D.J., Smartt C.T. (2013). Genome sequence analysis of dengue virus 1 isolated in Key West, Florida. PLoS ONE.

[57] Shin D., Civana A., Acevedo C., Smartt C.T. (2014). Transcriptomics of differential vector competence: West Nile virus infection in two populations of *Culex pipiens quinquefasciatus* linked to ovary development. BMC Genom..

[58] Alto B.W., Smartt C.T., Shin D., Bettinardi D., Malicoate J., Anderson S.L., Richards S.L. (2014). Susceptibility of Florida *Aedes aegypti* and *Aedes albopictus* to dengue viruses from Puerto Rico. J. Vector Ecol..

[59] Jupatanakul N., Sim S., Dimopoulos G. (2014). *Aedes aegypti* ML and Niemann-Pick type C family members are agonists of dengue virus infection. Dev. Comp. Immunol..

[60] Galat A. (1993). Peptidylproline cis-trans-isomerases: Immunophilins. Eur. J. Biochem..

[61] Bonner J.M., Boulianne G.L. (2017). Diverse structures, functions and uses of FK506 binding proteins. Cell. Signal..

[62] Bierer B.E., Mattila P.S., Standaert R.F., Herzenberg L.A., Burakoff S.J., Crabtree G., Schreiber S.L. (1990). Two distinct signal transmission pathways in T lymphocytes are inhibited by complexes formed between an immunophilin and either FK506 or rapamycin. Proc. Natl. Acad. Sci. USA.

[63] Tsujimoto Y., Finger L.R., Yunis J., Nowell P.C., Croce C.M. (1984). Cloning of the chromosome breakpoint of neoplastic B cells with the t(14;18) chromosome translocation. Science.

[64] Boyd J.M., Malstrom S., Subramanian T., Venkatesh L.K., Schaeper U., Elangovan B., D’Sa-Eipper C., Chinnadurai G. (1994). Adenovirus E1B 19 kDa and Bcl-2 proteins interact with a common set of cellular proteins. Cell.

[65] Li M.-T., Di W., Xu H., Yang Y.-K., Chen H.-W., Zhang F.-X., Zhai Z.-H., Chen D.-Y. (2013). Negative regulation of RIG-I-mediated innate antiviral signaling by SEC14L1. J. Virol..

[66] Conway M.J., Londono-Renteria B., Troupin A., Watson A.M., Klimstra W.B., Fikrig E., Colpitts T.M. (2016). *Aedes aegypti* D7 saliva protein inhibits dengue virus infection. PLoS Negl. Trop. Dis..

[67] Hoffmann J.A., Kafatos F.C., Janeway C.A., Ezekowitz R.A.B. (1999). Phylogenetic perspectives in innate immunity. Science.

[68] Kean J., Rainey S.M., McFarlane M., Donald C.L., Schnettler E., Kohl A., Pondeville E. (2015). Fighting arbovirus transmission: Natural and engineered control of vector competence in *Aedes* mosquitoes. Insects.

[69] Liang G., Gao X., Gould E.A. (2015). Factors responsible for the emergence of arboviruses; strategies, challenges and limitations for their control. Emerg. Microbes Infect..

[70] Behura S.K., Gomez-Machorro C., deBruyn B., Lovin D.D., Harker B.W., Romero-Severson J., Mori A., Severson D.W. (2014). Influence of mosquito genotype on transcriptional response to dengue virus infection. Funct. Integr. Genom..

[71] Behura S.K., Gomez-Machorro C., Harker B.W., deBruyn B., Lovin D.D., Hemme R.R., Mori A., Romero-Severson J., Severson D.W. (2011). Global cross-talk of genes of the mosquito *Aedes aegypti* in response to dengue virus infection. PLoS Negl. Trop. Dis..

[72] Shirane M., Nakayama K.I. (2002). Inherent calcineurin inhibitor FKBP38 targets Bcl-2 to mitochondria and inhibits apoptosis. Nat. Cell Biol..

[73] Hardwick J.M. (1998). Viral interference with apoptosis. Semin. Cell Dev. Biol..

[74] Saraiva R.G., Kang S., Simões M.L., Angleró-Rodríguez Y.I., Dimopoulos G. (2016). Mosquito gut antiparasitic and antiviral immunity. Dev. Comp. Immunol..

[75] Mims C.A., Day M.F., Marshall I.D. (1966). Cytopathic effect of Semliki Forest virus in the mosquito *Aedes aegypti*. Am. J. Trop. Med. Hyg..

[76] Bowers D.F., Coleman C.G., Brown D.T. (2003). Sindbis virus-associated pathology in *Aedes albopictus* (Diptera: Culicidae). J. Med. Entomol..

[77] Weaver S.C., Scott T.W., Lorenz L.H., Lerdthusnee K., Romoser W.S. (1988). Togavirus-associated pathologic changes in the midgut of a natural mosquito vector. J. Virol..

[78] Weaver S.C., Lorenz L.H., Scott T.W. (1992). Pathologic changes in the midgut of *Culex tarsalis* following infection with Western equine encephalomyelitis virus. Am. J. Trop. Med. Hyg..

[79] Vaidyanathan R., Scott T.W. (2006). Apoptosis in mosquito midgut epithelia associated with West Nile virus infection. Apoptosis.

[80] Liu B., Behura S.K., Clem R.J., Schneemann A., Becnel J., Severson D.W., Zhou L. (2013). P53-mediated rapid induction of apoptosis conveys resistance to viral infection in *Drosophila melanogaster*. PLoS Pathog..

