# Supplementary figures and images for: Homologs of Human Dengue-Resistance Genes, FKBP1B and ATCAY, Confer Antiviral Resistance in Aedes aegypti Mosquitoes
Source: Insects. 2019 Feb 2;10(2):46. doi: 10.3390/insects10020046 (PMC6409984; doi:10.3390/insects10020046)

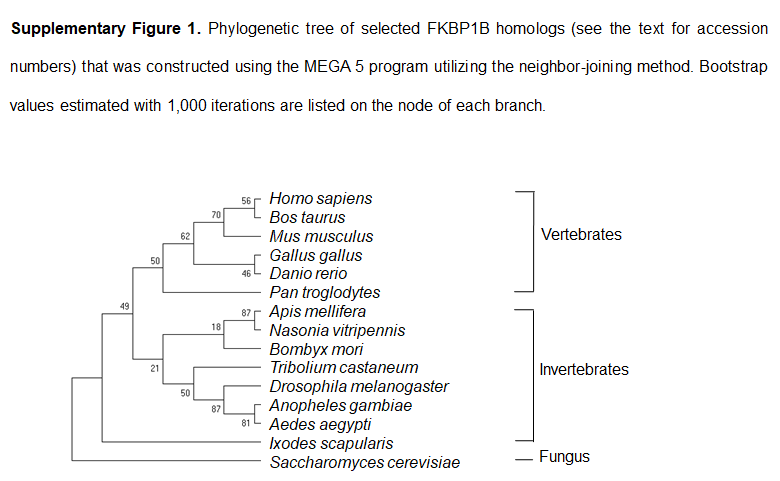

Supplement: Supplementary file 1 [file insects-10-00046-s001.zip › supplememtary FigureS1.tif]
